# Cholinergic α7 nAChR signaling suppresses SARS-CoV-2 infection and inflammation in lung epithelial cells

**DOI:** 10.1093/jmcb/mjad048

**Published:** 2023-07-25

**Authors:** Jing Wen, Jing Sun, Yanhong Tang, Jincun Zhao, Xiao Su

**Affiliations:** Unit of Respiratory Infection and Immunity, Institut Pasteur of Shanghai, Chinese Academy of Sciences, Shanghai 200031, China; CAS Key Laboratory of Molecular Virology and Immunology, Institut Pasteur of Shanghai, Chinese Academy of Sciences, Shanghai 200031, China; University of Chinese Academy of Sciences, Beijing 100049, China; State Key Laboratory of Respiratory Disease, National Clinical Research Centre for Respiratory Disease, Guangzhou Institute of Respiratory Health, The First Affiliated Hospital of Guangzhou Medical University, Guangzhou 510120, China; State Key Laboratory of Respiratory Disease, National Clinical Research Centre for Respiratory Disease, Guangzhou Institute of Respiratory Health, The First Affiliated Hospital of Guangzhou Medical University, Guangzhou 510120, China; State Key Laboratory of Respiratory Disease, National Clinical Research Centre for Respiratory Disease, Guangzhou Institute of Respiratory Health, The First Affiliated Hospital of Guangzhou Medical University, Guangzhou 510120, China; Unit of Respiratory Infection and Immunity, Institut Pasteur of Shanghai, Chinese Academy of Sciences, Shanghai 200031, China; CAS Key Laboratory of Molecular Virology and Immunology, Institut Pasteur of Shanghai, Chinese Academy of Sciences, Shanghai 200031, China; University of Chinese Academy of Sciences, Beijing 100049, China

**Dear Editor**,

Coronavirus disease 2019 (COVID-19), caused by severe acute respiratory syndrome coronavirus 2 (SARS-CoV-2) infection, has led to >6 million deaths and posed a huge threat to the global economy and public health. SARS-CoV-2 enters lung epithelial cells depending on the binding between SARS-CoV-2 S protein and the host receptor angiotensin-converting enzyme 2 (ACE2). In addition, *in-silico* studies indicated that both SARS-CoV and SARS-CoV-2 S glycoproteins can interact with the extracellular domain of α7 nicotinic acetylcholine receptor (nAChR). Given that α7 nAChR possesses anti-inflammatory properties and may interact with SARS-CoV-2 S protein, activating α7 nAChR-mediated cholinergic anti-inflammatory pathway might be an ideal therapeutic strategy for COVID-19. However, whether activation of α7 nAChR truly affects SARS-CoV-2 replication is still elusive.

In this study, human lung epithelial Calu3 cells were simultaneously treated with GTS-21 (a specific agonist of α7 nAChR) and infected with the SARS-CoV-2 pseudovirus containing the S protein as a surface capsid glycoprotein (hereafter referred to as SARS-CoV-2 pseudovirus). We found that activation of α7 nAChR significantly reduced S protein entry (Figure 1A). This finding was recapitulated in human colon epithelial Caco2 cells (Figure 1B). Next, Calu3 cells were simultaneously treated with GTS-21 and infected with wild-type SARS-CoV-2. We found that viral titers in supernatant were 124 times lower in the GTS-21-treated group than in the PBS-treated (control) group (Figure 1C and D), suggesting an anti-SARS-CoV-2 effect of α7 nAChR-mediated cholinergic signaling. Furthermore, immunofluorescence and real-time quantitative polymerase chain reaction (RT-qPCR) assays revealed significantly reduced ACE2 protein and mRNA levels, respectively, in Calu3 cells treated with GTS-21 and infected with SARS-CoV-2 pseudovirus (Figure 1E and F), suggesting that activation of α7 nAChR could disrupt SARS-CoV-2 entry by suppressing ACE2 expression.

**Figure 1:**
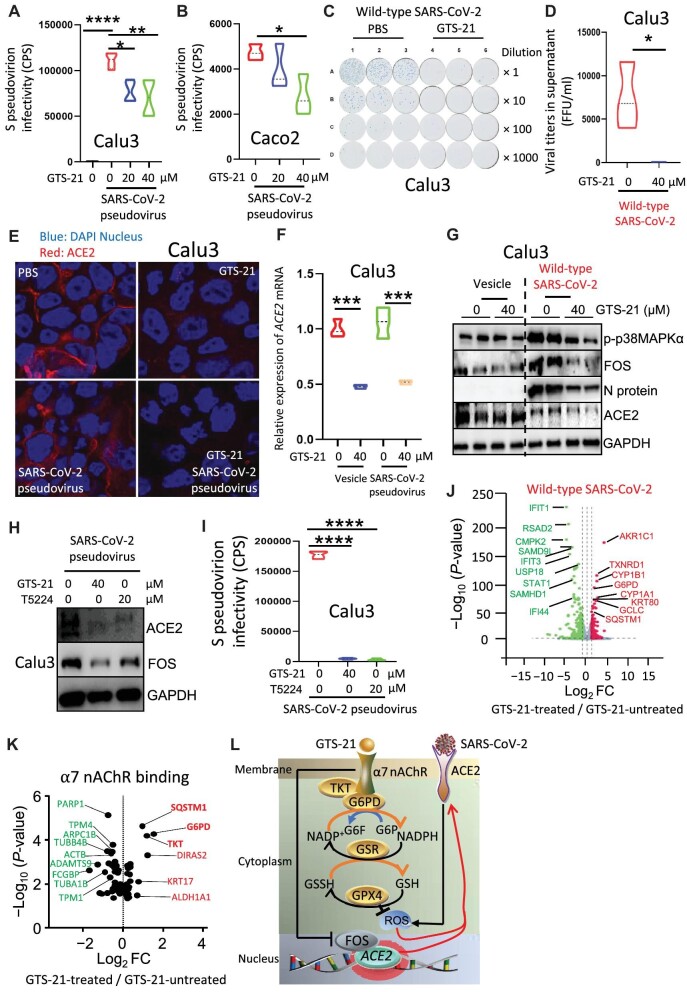
Cholinergic α7 nAChR signaling suppresses SARS-CoV-2 infection in lung epithelial cells. (**A** and **B**) Calu3 and Caco2 cells were simultaneously infected with SARS-CoV-2 pseudovirus and treated with different concentrations of GTS-21 for 24 h. The Viral infectivity was analyzed by luciferase assays. *P < 0.05, **P < 0.01, ****P < 0.0001, *n* = 3–4 in each group, one-way ANOVA. CPS, counts per second. (**C** and **D**) Calu3 cells were simultaneously infected with wild-type SARS-CoV-2 (0.02 MOI) and treated with GTS-21 for 24 h. Viral titers in cell supernatant were detected by a foci-forming assay. *P < 0.05, *n* = 3 in each group, unpaired *t*-test. (**E** and **F**) Calu3 cells were simultaneously infected with SARS-CoV-2 pseudovirus and treated with GTS-21 for 24 h. ACE2 protein and mRNA expression levels were detected by immunofluorescence and RT-qPCR, respectively. ***P < 0.001, *n* = 3 in each group, one-way ANOVA. (**G**) Calu3 cells were simultaneously infected with wild-type SARS-CoV-2 (0.02 MOI) and treated with GTS-21 for 24 h. The protein expression levels of p-p38MAPKα, FOS, the viral N protein, and ACE2 were detected by western blotting. (**H** and **I**) Calu3 cells were treated with GTS-21 and T5224 for 4 h and then infected with SARS-CoV-2 pseudovirus for 24 h. (**H**) The protein expression levels of FOS and ACE2 were detected by western blotting. (**I**) The viral infectivity was analyzed by luciferase assays. ****P < 0.0001, *n* = 3 in each group, one-way ANOVA. (**J**) Calu3 cells were infected with wild-type SARS-CoV-2 and treated with or without GTS-21. Volcano plot analysis was performed with upregulated and downregulated genes in GTS-21-treated vs. GTS-21-untreated cells. *P < 0.05, *n* = 3 in each group, unpaired *t*-test. FC, fold change. (**K**) A total of 178 proteins were identified to interact with α7 nAChR in Supplementary Figure S4. Genes for 145 such proteins were found in our RNAseq database and subject to volcano plot analysis. *P < 0.05, *n* = 3 in each group, unpaired *t*-test. (**L**) A hypothetical model showing that activation of α7 nAChR suppresses SARS-CoV-2 infection. The binding between SARS-CoV-2 S protein and ACE2 triggers ROS to promote SARS-CoV-2 S protein entry. Activation of α7 nAChR recruits G6PD and TKT to enhance the anti-ROS capacity and suppress SARS-CoV-2 S protein entry. Moreover, activation of α7 nAChR compromises the binding of FOS to the ACE2 promoter, thus suppressing *ACE2* expression and SARS-CoV-2 S protein entry.

Then, RNA sequencing (RNAseq) was performed with Calu3 cells treated with or without GTS-21 and infected with or without wild-type SARS-CoV-2 (BGI BIG DATABASE, see Supplementary Materials and methods). Data analysis revealed that activation of α7 nAChR inhibited the expression of 39 viral defense response genes (Supplementary Figure S1A–C), suggesting that viral replication might be disrupted. Activation of α7 nAChR also significantly reduced the mRNA level of *FOS*, a member of the AP-1 family (Supplementary Figure S2A–C), and suppressed both ACE2 and FOS protein levels in SARS-CoV-2 pseudovirus-infected Calu3 cells (Supplementary Figure S2D). Consistently, activation of α7 nAChR significantly reduced the protein levels of p-p38MAPKα, FOS, the viral nucleocapsid (N protein), and ACE2 in wild-type SARS-CoV-2-infected Calu3 cells (Figure 1G). In addition, *FOS* knockdown in Caco2 cells markedly reduced ACE2 mRNA and protein levels (Supplementary Figure S2E and F) and SARS-CoV-2 pseudovirus infectivity (Supplementary Figure S2G). These findings suggest that FOS is required for ACE2 expression and SARS-CoV-2 S protein entry. Next, Calu3 cells were treated with T5224 (a compound that inhibits FOS binding to the ACE2 promoter) and then infected with SARS-CoV-2 pseudovirus. Western blotting and luciferase assays demonstrated that the inhibition of FOS binding reduced ACE2 protein level (Figure 1H) and SARS-CoV-2 pseudovirus infectivity (Figure 1I), respectively. These findings suggest that activation of α7 nAChR reduces FOS expression levels to suppress ACE2 expression and disrupt SARS-CoV-2 S protein entry.

The binding between SARS-CoV-2 S protein and ACE2 can induce reactive oxygen species (ROS) production (Li et al., 2021). Thus, we examined whether activation of α7 nAChR affects ROS production by using a dichlorodihydrofluorescein diacetate (DCFH-DA) fluorescent probe and found that activation of α7 nAChR significantly reduced ROS levels in SARS-CoV-2 pseudovirus-infected Calu3 cells (Supplementary Figure S3A and B). Meanwhile, Caco2 cells were pretreated with H_2_O_2_ and then infected with SARS-CoV-2 pseudovirus. H_2_O_2_ significantly increased SARS-CoV-2 infectivity (Supplementary Figure S3C), suggesting that ROS mediate SARS-CoV-2 S protein entry. To determine the anti-oxidative mechanism of GTS-21, we performed volcano plot analysis with the RNAseq data and identified 164 genes upregulated and 448 genes downregulated in GTS-21-treated wild-type SARS-CoV-2-infected Calu3 cells compared to GTS-21-untreated infected cells. Activation of α7 nAChR significantly increased the expression levels of anti-oxidative genes, including *TXNRD1* (maintaining redox homoeostasis), *G6PD* (maintaining a normal NADPH/NADP ratio that in turn regulates glutathione biosynthesis), *GCLC* (the first rate-limiting enzyme of glutathione synthesis), and *SQSTM1* (Figure 1J).

To further elucidate how α7 nAChR mediates anti-oxidative effects, anti-α7 nAChR antibodies were used to pull down its binding proteins. Mass spectrometry analysis identified 178 proteins that could bind to α7 nAChR. The gene transcripts encoding 145 of these proteins were found in our RNAseq database. Through volcano plot analysis, we found that the mRNA levels of anti-oxidative genes, including *G6PD, TKT* (decreasing glucose flux into glycolysis and increasing glutathione synthesis), and *SQSTM1*, were significantly increased in GTS-21-treated SARS-CoV-2-infected Calu3 cells (Figure 1K; Supplementary Figure S4A and B). These findings suggest that activation of α7 nAChR may recruit and promote G6PD and TKT to execute their anti-oxidative activities.

Further analysis of the RNAseq data indicated that activation of α7 nAChR increased the mRNA levels of *GPX4* and *GPX2* (Supplementary Figure S5A–C), which play pivotal roles in maintaining glutathione function and inhibiting ferroptosis, as well as *GCLC* and *GCLM* (genes related to glutathione biosynthesis) but reduced the mRNA level of *LAP3* (a glutathione proteolysis gene) (Supplementary Figure S5A–C). These findings suggest that activation of α7 nAChR is associated with increased glutathione production and the anti-oxidative properties.

Many studies and clinical trials have reported that vagus nerve stimulation can activate α7 nAChR and suppress the cytokine storm in the lung of COVID-19 patients (Bonaz et al., 2020; Azabou et al., 2021). It was reported that local electric stimulation prevents SARS-CoV-2 S protein from binding to ACE2 in the lung epithelium (Kipshidze et al., 2020). In our study, activation of α7 nAChR also significantly reduced the mRNA levels of cytokine storm-associated genes (e.g. *CXCL1, CCL20, CXCL8*, and *CXCL5*) in lung epithelial cells (Supplementary Figure S6A–C), confirming that activation of α7 nAChR suppresses the cytokine storm in lung epithelial cells.

Previous studies showed that activation of α7 nAChR could attenuate lung injury through downregulating HMGB1 and NF-κB signaling (Kox et al., 2011; Sitapara et al., 2020a, b). Similar to that observed for the mRNA levels of α7 nAChR (Supplementary Figure S7A–C; Reghunathan et al., 2005), SARS-CoV-2 did not affect *Hmgb1* mRNA levels in mouse lungs and activation of α7 nAChR did not affect *HMGB1* mRNA levels in SARS-CoV-2-infected Calu3 cells (Supplementary Figure S7D and E). Meanwhile, in SARS-CoV-2-infected mouse lungs, *Nfkbid, Nfkbie, Nfkb2, Nfkbiz*, and *Nfkbil1* were upregulated (Supplementary Figure S7F), suggesting the activated NF-κB signaling. In SARS-CoV-2-infected Calu3 cells, activation of α7 nAChR significantly upregulated two NF-κB inhibitors *NFKBIL1* and *NFKBIB* (Supplementary Figure S7G), suggesting that activation of α7 nAChR may inhibit NF-κB. FOS is a member of the AP-1 family, which can be activated by NF-κB (Hsieh et al., 2010). Compared to SARS-CoV-2-infected *Chrna7^+/+^*mice, SARS-CoV-2-infected *Chrna7^−^^/^^−^* mice showed higher *Fos* and *Cxcl2* mRNA levels (Supplementary Figure S8A) and severe inflammation (Supplementary Figure S8B) in the lung, suggesting that activation of α7 nAChR could suppress FOS expression and lung inflammation during SARS-CoV-2 infection. Therefore, activation of α7 nAChR disturbs SARS-CoV-2-induced NF-κB–FOS signaling.

Taken together, the binding between SARS-CoV-2 S protein and ACE2 triggers ROS, which promote SARS-CoV-2 S protein entry. Activation of α7 nAChR likely increases glutathione biosynthesis and reduces ROS-mediated SARS-CoV-2 S protein entry. Moreover, activation of α7 nAChR compromises the binding of FOS to the ACE2 promoter and therefore suppresses *ACE2* expression and SARS-CoV-2 S protein entry (Figure 1L). Activation of α7 nAChR also suppresses inflammation in SARS-CoV-2-infected lung epithelial cells. These findings prompt us to target lung epithelial α7 nAChR to curb COVID-19 by reducing viral replication and proinflammatory responses. Electric stimulation of the vagus nerve to increase the excitability or administration of α7 nAChR agonists into the airways might be promising strategies for combating this deadly disease.

*This work was supported by grants from the National Natural Science Foundation of China (82241042, 81730001, 91942305, and 81970075), the National Key Research and Development Program of China (2022YFC2304700), the Science and Technology Commission of Shanghai Municipality (20DZ2261200), and the Innovative Research Team of High-level Local Universities in Shanghai (SHSMU-ZDCX20210602). X.S. and J.Z. conceived and designed the study. J.W. conducted pseudovirus entry assays, RT-qPCR, western blotting, immunofluorescence experiments, mass spectrometry, and RNAseq. J.S. conducted wild-type SARS-CoV-2 infection experiments. J.Z. provided Calu3 and Caco2 cell lines, SARS-CoV-2 pseudovirus, and the P3 facility for performing wild-type SARS-CoV-2 infection experiments. J.W., J.S., Y.T., J.Z., and X.S. prepared the figures. J.W., J.C., and X.S. wrote the manuscript. RNAseq *was performed* using the DNBSEQ platform (BGI BIG DATABASE, https://biosys.bgi.com). The transcriptome data, including 18225 genes, can be downloaded from the DNBSEQ platform and will be delivered upon request.]*

## Supplementary Material

mjad048_Supplemental_File
