# Supplementary material for: Cholinergic α7 nAChR signaling suppresses SARS-CoV-2 infection and inflammation in lung epithelial cells
Source: J Mol Cell Biol. 2023 Jul 25;15(7):mjad048. doi: 10.1093/jmcb/mjad048 (PMC10901198; doi:10.1093/jmcb/mjad048)

Cholinergic-α7 nAChR signaling suppresses SARS-CoV-2 infection and inflammation in lung epithelial cells

Jing Wen, Jing Sun, Yanhong Tang, Jincun Zhao, Xiao Su

Supplementary information

Materials and Methods

Antibodies and compounds

Anti-ACE2 (ab272500, Abcam), anti-FOS (66590-1-Ig, Proteintech), anti-SARS-CoV-2 nucleocapsid (ab271180, Abcam), anti-GAPDH (M20006; Abmart), Rhodamine (TRITC) AffiniPure Goat Anti-Rabbit IgG (Jackson Immunoresearch). GTS-21 (ab120560, Abcam), T5224 (T5416, Topscience).

Cells

HEK293T cell line were cultured in Dulbecco's Modified Eagle Medium (DMEM) (Gibco) supplemented with 10% fetal bovine serum (Gibco), 100 U/ml penicillin, and 100 μg/ml of streptomycin (Invitrogen) at 37°C under 5% CO_2. Calu3 cell line were cultured in Minimum Essential Medium (MEM) (Gibco) supplemented with 20% fetal bovine serum (Gibco), 100 U/ml penicillin, and 100 μg/ml of streptomycin (Invitrogen) at 37°C under 5% CO_2. Caco2 cell line were cultured in Eagle's minimum essential medium (MEM) (WISENT) supplemented with 20% fetal bovine serum (Gibco), 100 U/ml penicillin, and 100 μg/ml of streptomycin (Invitrogen) at 37°C under 5% CO_2.

Animals

α7nAChR-deficient mice (C57BL/6 background, B6.129S7-*Chrnα7*tm1Bay, number 003232) were purchased from the Jackson Laboratory (Bar Harbor, ME, USA. The mice were housed at 22°C on a 12 h light/dark cycle and with free access to sufficient food

and water. Anesthesia was conducted with pentobarbital sodium (50 mg/kg) injected intraperitoneally (ip). The animal studies were approved by the Committees on Animal Research of the Institut Pasteur of Shanghai, Chinese Academy of Sciences. The mice were instilled with SARS-CoV-2 (African strain) into the nasal cavity at a dose of 5×10^4 FFU. All SARS-CoV-2 infection experiments were performed in a biosafety level-3 laboratory (Guangzhou Institute of Respiratory Health, Guangzhou, Guangdong China).

SARS-CoV-2 pseudovirus production and virus entry assay

HEK293T cells were co-transfection with pSPAX2, pLenti-GFP-luc and pCDNA3.1-SARS-CoV-2-S by Lipo2000. The supernatants were harvested at 48 h post transfection, filtered with 0.45 μm filter, and centrifuged at 1000 × g for 5 min to remove cell debris. To infect cells with pseudovirions, cells were seeded into 24-well plates and inoculated with 500 μl media containing pseudovirions. After 24 or 48 h, cells were lysed with 100 μl lysis buffer (promega) for 30 min on ice. The entry efficiency was measured by quantification of the luciferase activity (promega).

Focus forming assay (FFA)

Vero E6 cells were seeded in 96-well plates one day before infection. Virus cultures were serially diluted and used to inoculate Vero E6 cells at 37°C for 1 h. Inocula were then removed before adding 125 μl 1.6% carboxymethylcellulose warmed to 37°C per well. After 24 h, cells were fixed with 4% paraformaldehyde and permeabilized with 0.2% Triton X-100. Cells were then incubated with a rabbit anti-SARS-CoV-2 nucleocapsid protein polyclonal antibody (Cat. No.: 40143-T62, Sino Biological, Inc. Beijing), followed by an HRP-labeled goat anti-rabbit secondary antibody (Cat. No.: 109-035-088, Jackson ImmunoResearch Laboratories, Inc. West Grove, PA). The foci were visualized by TrueBlue Peroxidase Substrate (KPL, Gaithersburg, MD), and counted with an ELISPOT reader (Cellular Technology Ltd. Cleveland, OH). Viral titers were calculated as FFU per ml or per gram tissue.

Real-time (RT-) qPCR

Total cellular RNA was extracted by TRIzol reagent (Invitrogen), and reverse transcribed into cDNA using a reverse transcriptase kit (Tiangen, Beijing, China). Real-time PCR was performed using the Thunderbird SYBR qPCR Mix (11203ES08; Yeasen) on the ABI QuantStudio 6 flex Real-Time PCR system. The primers were used as follows,

GAPDH: forward, 5′-ATCCCATCACCATCTTCCAGG-3′ and reverse, 5′-CCTTCTCCATGGTGG TGAAGA C-3′;

Human *ACE2*: forward, 5′-CAAGAGCAAACGGTTGAACAC-3′ and reverse, 5′-CCAGAGCCTCTCATTGTAGTCT-3′;

Human *FOS*: forward, 5′- GGGGCAAGGTGGAACAGTTAT-3′ and reverse, 5′-CCGCTTGGAGTGTATCAGTCA-3′;

Mouse *Fos*: forward 5′-CGGGTTTCAACGCCGACTA-3′ and reverse, 5′-TTGGCACTAGAGACGGACAGA3′;

Mouse *CXcl2*: forward 5′-GCTGTCAATGCCTGAAG-3′ and reverse, 5′-GGCGTCACACTCAAGCTCT-3′;

COVID19-N nucleocapsid: forward 5′-CGAACTTCTCCTGCTAGAATGG-3′ and reverse, 5′-GTGACAGTTTGGCCTTGTTG-3′.

RNA-Seq and RNA-Seq data analysis

The Calu3 cells (epithelial cells isolated from lung tissue) were pretreated with either PBS or GTS-21 (40 μM) for 30 min, then infected with or without SARS-CoV-2 virus (African strain). The cells were harvested at 24 h after infection. Therefore, we had 4 groups: PBS+PBS, GTS-21+PBS, PBS+SARS-CoV-2, and GTS-21+SARS-CoV-2. Each group had 3 samples.

The total RNA was extracted using TRIzol reagent (Invitrogen). After quality test, the total RNA of each sample was sequenced using the DNBSEQ platform (BGI BIG DATABASE (https://biosys.bgi.com/#/report/mrna/expression). Data were analyzed in online analysis software Dr. Tom and STRING online (https://string-db.org). GeneCards (the Human Gene Database) (https://www.genecards.org) was used to search genes with known functions.

Lung histology

Hematoxylin and eosin (H&E) staining of lungs was conducted as described previously in our laboratory.

Confocal microscopy

Cells were fixed in 4% formaldehyde and permeabilized with 0.1% Triton-X 100. Then, the cells were blocked with PBS containing 5% BSA for 30 min at room temperature. Further, the cells were stained with ACE2 antibody, followed by secondary Rhodamine (TRITC)-labeled anti-rabbit IgG antibodies (Jackson Immunoresearch) at room temperature. The nucleus was labeled with DAPI. Slides were imaged on a laser-scanning confocal microscope (Olympus FV-1200). Images were quantified by Image J Pro.

Reactive oxygen species (ROS) assay

ROS were detected by using ROS Assay Kit-Highly Sensitive DCFH-DA kit (DOJINDO LABORATORIES). Calu3 cells were washed twice with HBSS buffer. Add DCFH-DA Dye probe to the cells and mixed completely, cells were incubated at 37°C for 30 min. Then wash cells for twice with HBSS buffer and observe ROS by fluorescence microscope.

Western blotting

Cells were lysed for 30 min at 4°C in lysis buffer (Beyotime). After centrifugation for 10 min at 12,000 × g, supernatant was boiled in loading buffer and analyzed by SDS-PAGE. Specific primary antibodies were used, followed by horseradish peroxidase-conjugated goat anti-mouse IgG or goat anti-rabbit IgG (Jackson Immunoresearch) as the secondary antibodies.

Immunoprecipitation-Mass Spectrometry

A549 cells treated with or without 40 μM GTS-21 for 48 h, cells were collected and lysed in IP lysis buffer (Beyotime) for 30 min on ice. After centrifugation for 10 min at 12,000 × g, the supernatants were incubated with 5 μg anti- α7 nAChR (sc-58607, Stan Cruz) antibody at 4°C overnight. 25 μl Protein G-labeled Dynabeads (Thermo Scientific)

were added into each sample at 4°C for 6 h. Dynabeads were washed 3 times with RIPA buffer. Proteins were eluted by boiling 10 min with loading buffer. The immunoprecipitates were separated by SDS-PAGE, each pull-down sample was run into the separation gel. We cut off the whole band as one sample and subjected it to in-gel trypsin digestion and Mass Spectrometry analysis (Performed by Orbitrap Velos Pro, Shanghai Institute of Nutrition and Health, Chinese Academy of Sciences).

Statistical analysis

Statistics were calculated using GraphPad Prism software (GraphPad, San Diego, CA). An unpaired t-test was used unless there were multiple comparisons, in which case we used one-way ANOVA with a post hoc Bonferroni test (with a significance level of $p < 0.05$). The results are shown as mean ± SD.

Supplementary figures and legends

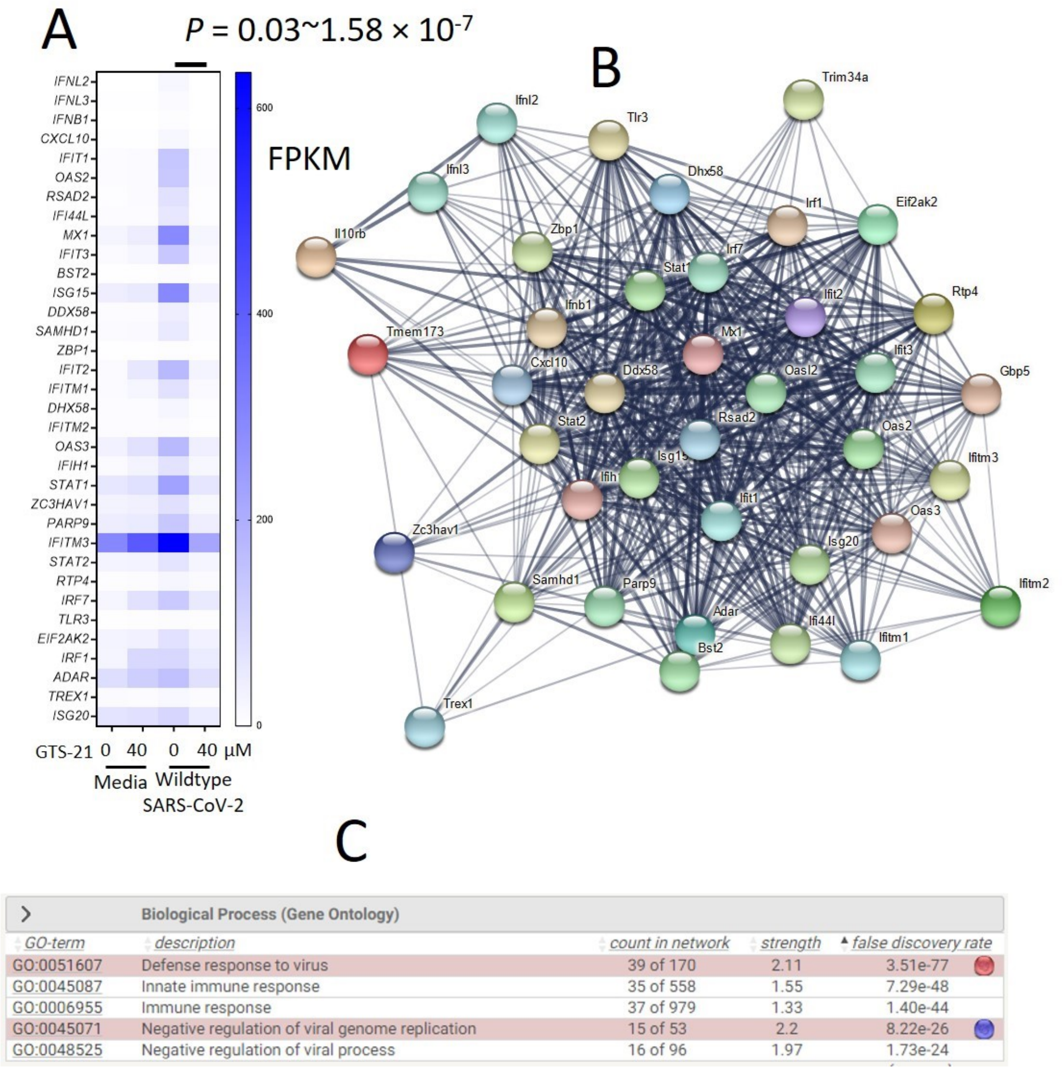

GO-term	description	count in network	strength	false discovery rate
GO:0051607	Defense response to virus	39 of 170	2.11	3.51e-77
GO:0045087	Innate immune response	35 of 558	1.55	7.29e-48
GO:0006955	Immune response	37 of 979	1.33	1.40e-44
GO:0045071	Negative regulation of viral genome replication	15 of 53	2.2	8.22e-26
GO:0048525	Negative regulation of viral process	16 of 96	1.97	1.73e-24

Supplementary Figure S1. Analysis of downregulated viral defense response genes in RNAseq data from GTS-21 treated SARS-CoV-2 infected Calu3 cells. A Hotmap of enriched genes related to viral defense responses in PBS or GTS-21 treated Calu3 cells infected with or without wild SARS-CoV-2. N = 3 in each group, P values are indicated between PBS + SARS-CoV-2 and GTS-21 + SARS-CoV-2 groups. Unpaired t test. **B-C** Protein interaction network and KEGG analysis of enriched genes related to viral defense responses in PBS or GTS-21 treated Calu3 cells infected with or without wild SARS-CoV-2.

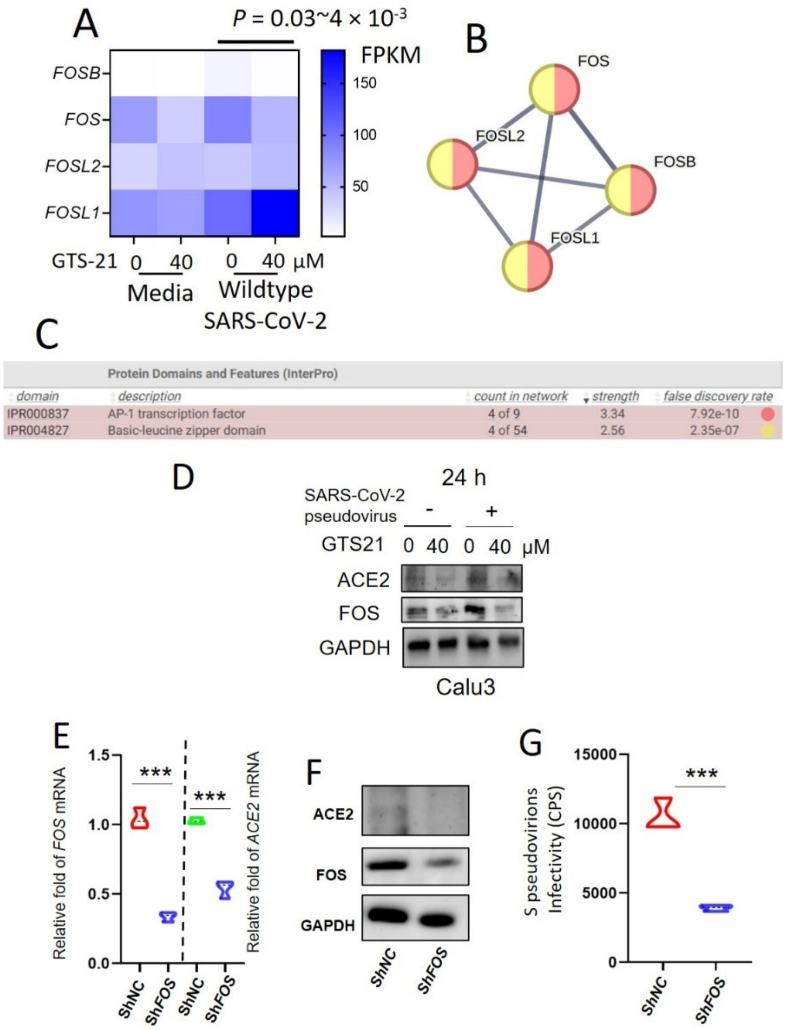

Supplementary Figure S2. Activation of α7 nAChR suppresses ACE2 expression depending on FOS. **A** Hotmap of enriched genes of *FOS* family in PBS or GTS-21 treated Calu3 cells infected with or without wild SARS-CoV-2 N = 3 in each group, P values are indicated between PBS + SARS-CoV-2 and GTS-21 + SARS-CoV-2 groups. Unpaired t test. **B-C** Protein interaction network and GO analysis of enriched *FOS* family genes in PBS or GTS-21 treated Calu3 cells infected with or without wild SARS-CoV-2. **D** Calu3 cells were pretreated with GTS-21 for 24 h and infected with pseudovirus for another 24 h, the expression levels of FOS and ACE2 were detected by western blotting. **E-F** In Caco2 cells, the endogenous *FOS* was knocked down with lentiviruses containing specific shRNA. The mRNA and protein expression levels of FOS and ACE2 were detected by RT-qPCR and western blotting. N = 3 in each group, ***P < 0.01 unpaired-t test. **G** the negative control and *FOS* knockdown cells were infected

with S pseudovirus, the infectivity was analyzed by luciferase assay. N = 3 in each group, **P < 0.01 unpaired-t test.

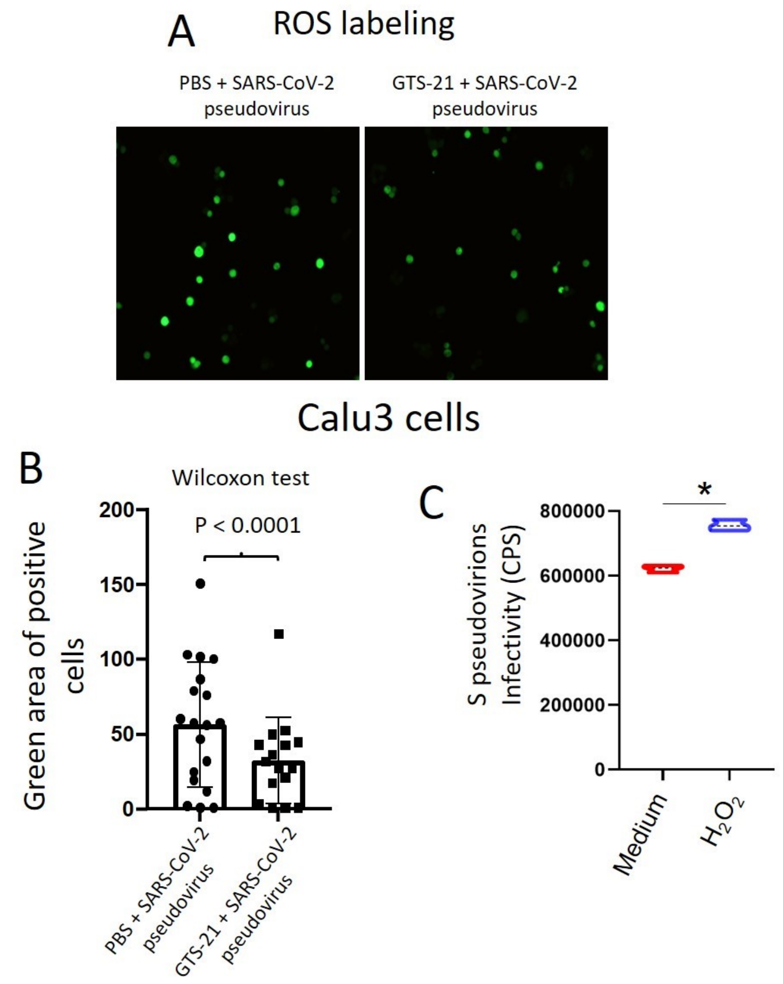

Supplementary Figure S3. Activation of α7 nAChR suppresses ROS and SARS-CoV-2 S protein entry. A-B Calu3 cells were infected with pseudovirus and treated with GTS-21 for 6 h, intracellular ROS was labeled by DCFH-DA fluorescent probes, ROS level was observed by fluorescence microscope. Green positive areas were analyzed by Image-pro Plus software. Experiments were repeated 3 times. P values are indicated between compared groups. Wilcoxson test. **C** Caco2 cells were pretreated with H_2O_2 for 4 h and then infected with pseudovirus for 24 h, virus infection level was analyzed by luciferase assay. N = 3 in each group, *P < 0.05, unpaired-t test.

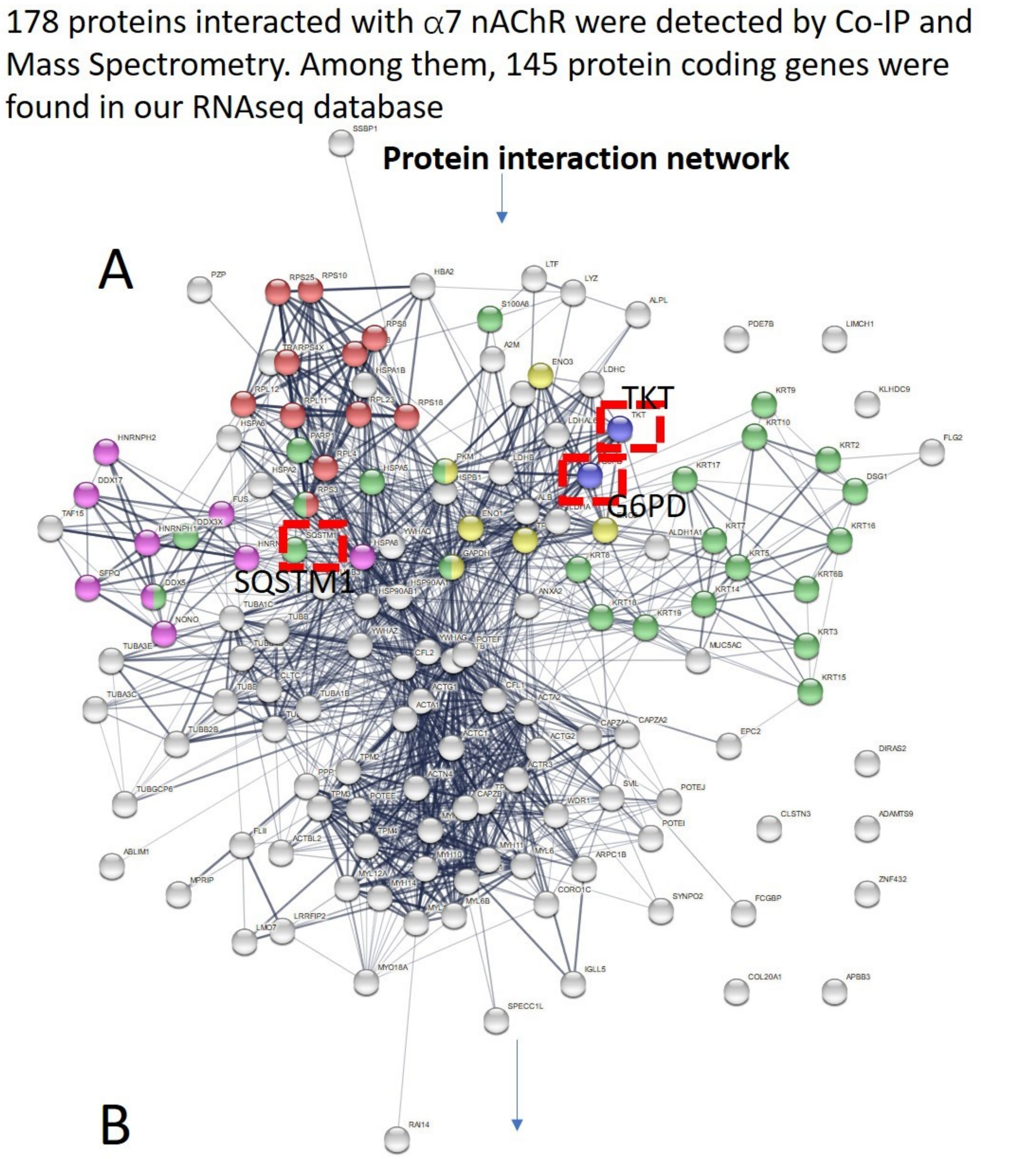

GO-term	description	count in network	strength	false discovery rate
GO:0019083	Viral transcription	11 of 115	1.11	9.52e-07
GO:0061621	Canonical glycolysis	6 of 27	1.48	2.78e-05
GO:0012501	Programmed cell death	24 of 1054	0.49	0.00013
GO:0000398	mRNA splicing, via spliceosome	9 of 294	0.62	0.0212
GO:0019322	Pentose biosynthetic process	2 of 4	1.83	0.0352

Supplementary Figure S4. Analysis of 145 α7 nAChR-binding proteins incorporated with RNAseq data from GTS-21 treated SARS-CoV-2 infected Calu3 cells. A Protein interaction network in 145 α7 nAChR-binding proteins. **B** GO analysis of genes of 145

α7 nAChR-binding proteins. N = 3 in each group, $P < 0.05$ is considered as a significant difference in compared groups when identified the differentially expressed genes. Unpaired-t test.

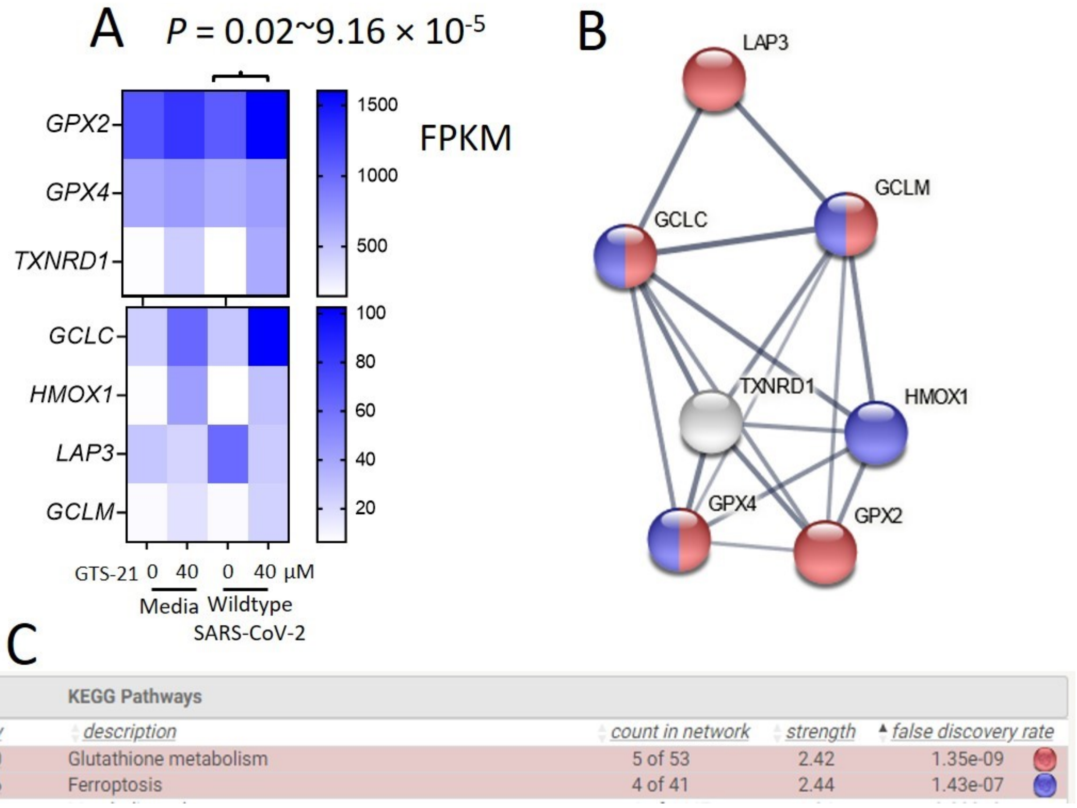

Supplementary Figure S5. Analysis of glutathione metabolism genes in RNAseq data from GTS-21 treated SARS-CoV-2 infected Calu3 cells. A Hotmap of enriched genes related to glutathione metabolism in PBS or GTS-21 treated Calu3 cells infected with or without wild SARS-CoV-2. N = 3 in each group, P values are indicated between PBS + SARS-CoV-2 and GTS-21 + SARS-CoV-2 groups. Unpaired t test. **B-C** Protein interaction network and KEGG analysis of enriched genes related to glutathione metabolism in PBS or GTS-21 treated Calu3 cells infected with or without wild SARS-CoV-2.

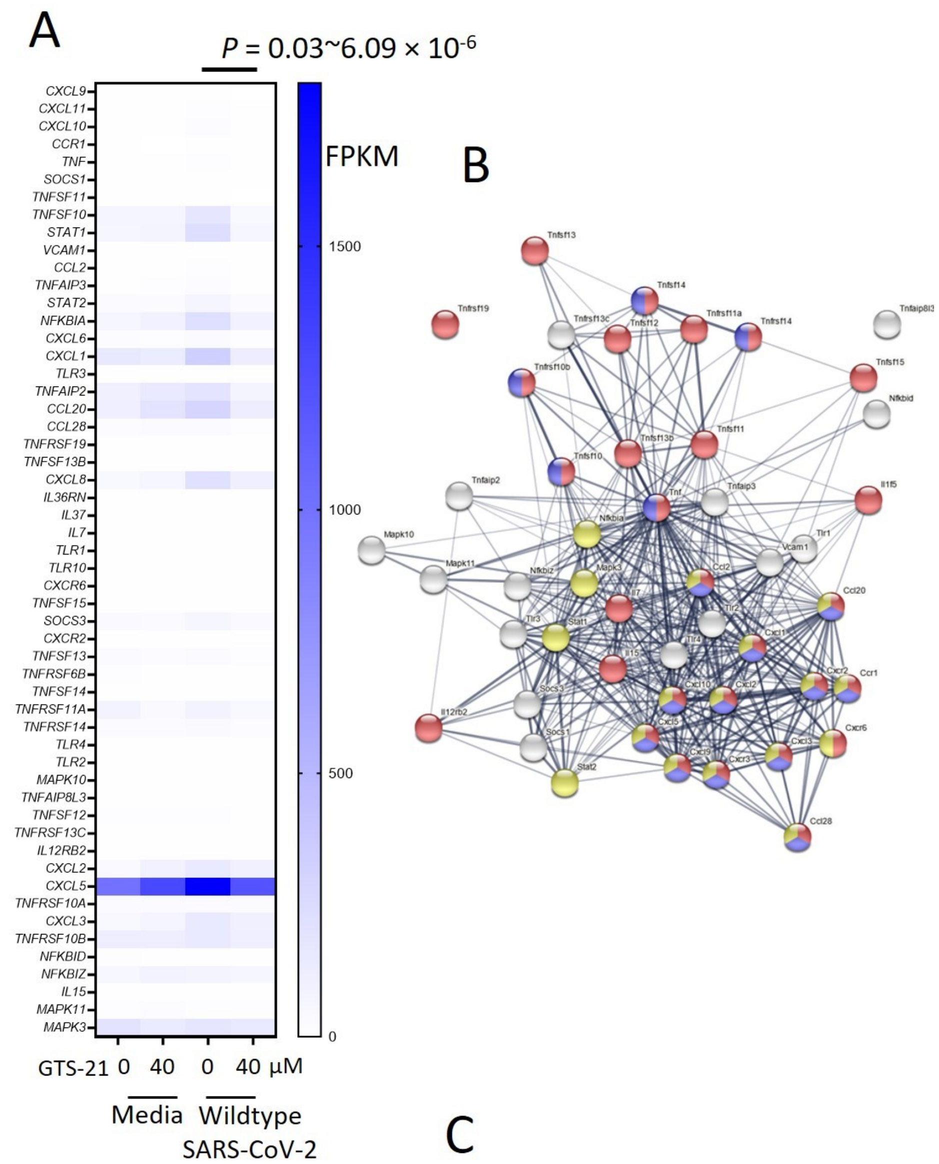

C

pathway	description	count in network	strength	false discovery rate
mmu04060	Cytokine-cytokine receptor interaction	29 of 279	1.68	1.21e-39
mmu04061	Viral protein interaction with cytokine and cytokine receptor	17 of 87	1.95	4.33e-26
mmu04668	TNF signaling pathway	16 of 112	1.82	1.30e-22
mmu04062	Chemokine signaling pathway	17 of 184	1.63	2.77e-21

Supplementary Figure S6. Analysis of cytokine storm genes in RNAseq data from GTS-21 treated SARS-CoV-2 infected Calu3 cells. A Hotmap of enriched genes related to cytokine storm in PBS or GTS-21 treated Calu3 cells infected with or without wild SARS-CoV-2. N = 3 in each group, P values are indicated between PBS + SARS-CoV-2 and GTS-21 + SARS-CoV-2 groups. Unpaired t test. **B-C** Protein interaction network and KEGG analysis of enriched genes related to cytokine and chemokine in PBS or GTS-21 treated Calu3 cells infected with or without wild SARS-CoV-2.

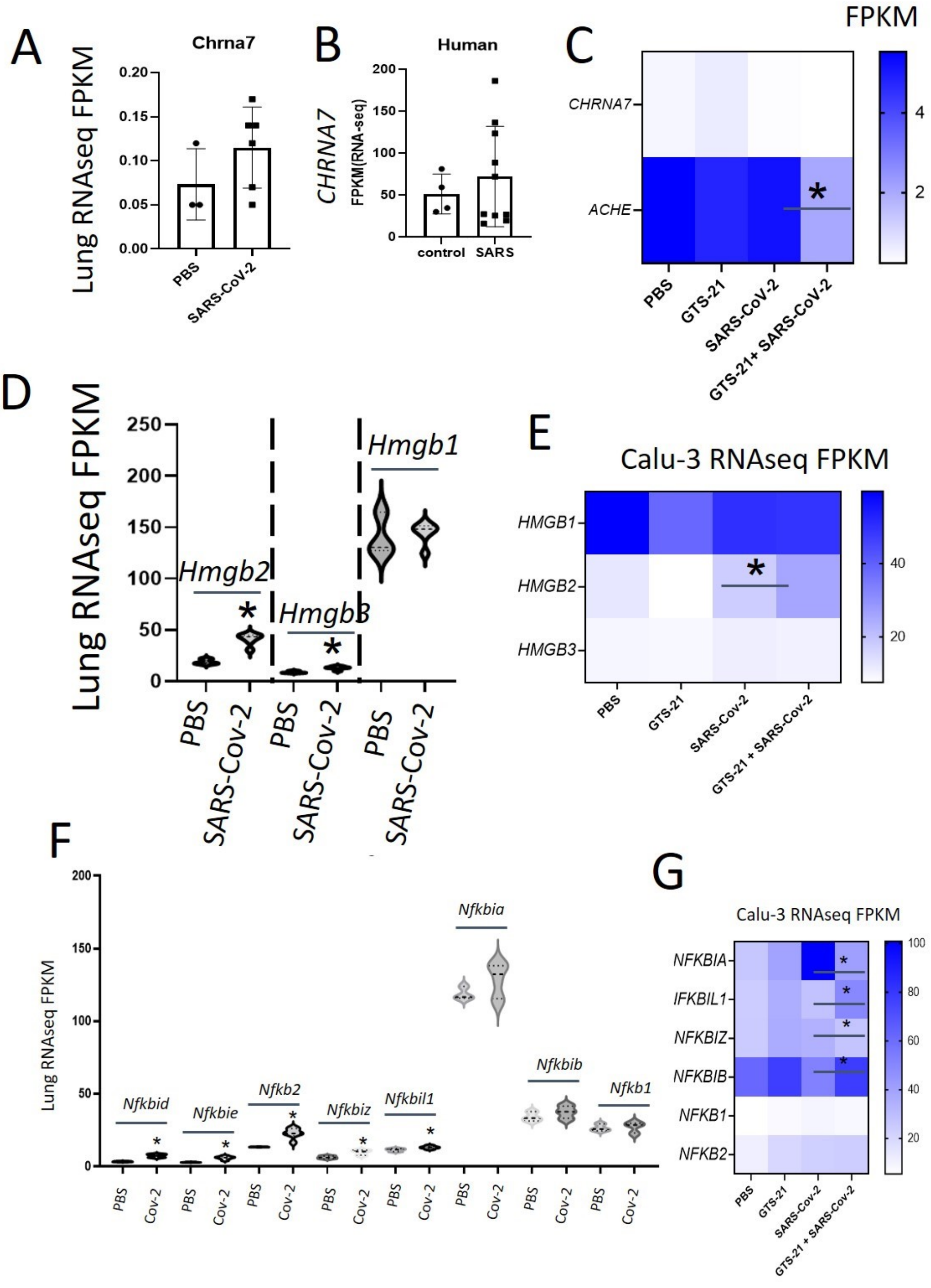

Supplementary Figure S7. Activation of α7 nAChR, which regulates SARS-CoV-2 infection, involves upregulation of α7 nAChR, acetylcholine hydrolysis, HMGB and NF-kB activation. A-B. α7 nAChR expression at mRNA levels in SARS-CoV-2-infected mouse lungs and human peripheral mononuclear cells isolated from SRAS patients. We analyzed GEO DataSets in Pubmed

(https://www.ncbi.nlm.nih.gov/sites/GDSbrowser?acc=GDS1028). Expression profiling of peripheral blood mononuclear cells (PBMC) from 10 adult patients with severe acute respiratory syndrome (SARS) was presented. **C** Hotmap of *CHRNA7* and *ACHE* in PBS or GTS-21 treated Calu3 cells infected with or without wild SARS-CoV-2. N = 3 in each group, *P < 0.05 between PBS + SARS-CoV-2 and GTS-21 + SARS-CoV-2 groups. Unpaired t test. **D** Gene expression of *Hmgb*-related genes in in SARS-CoV-2-infected mouse lungs. N=3 in PBS and N = 6 in SARS-CoV-2 infected mice. Unpaired t test. **E** Hotmap of *HMGB1, HMGB2* and HMGB3 in PBS or GTS-21 treated Calu3 cells infected with or without wild SARS-CoV-2. N = 3 in each group, *P < 0.05 between PBS + SARS-CoV-2 and GTS-21 + SARS-CoV-2 groups. Unpaired t test. **F** Gene expression of *Nfkb*-related genes in in SARS-CoV-2-infected mouse lungs. N = 3 in PBS and N = 6 in SARS-CoV-2 infected mice. Unpaired t test. **G** Hotmap of *NFKB* related genes in PBS or GTS-21 treated Calu3 cells infected with or without wild SARS-CoV-2. N = 3 in each group, *P < 0.05 between PBS + SARS-CoV-2 and GTS-21 + SARS-CoV-2 groups. Unpaired t test.

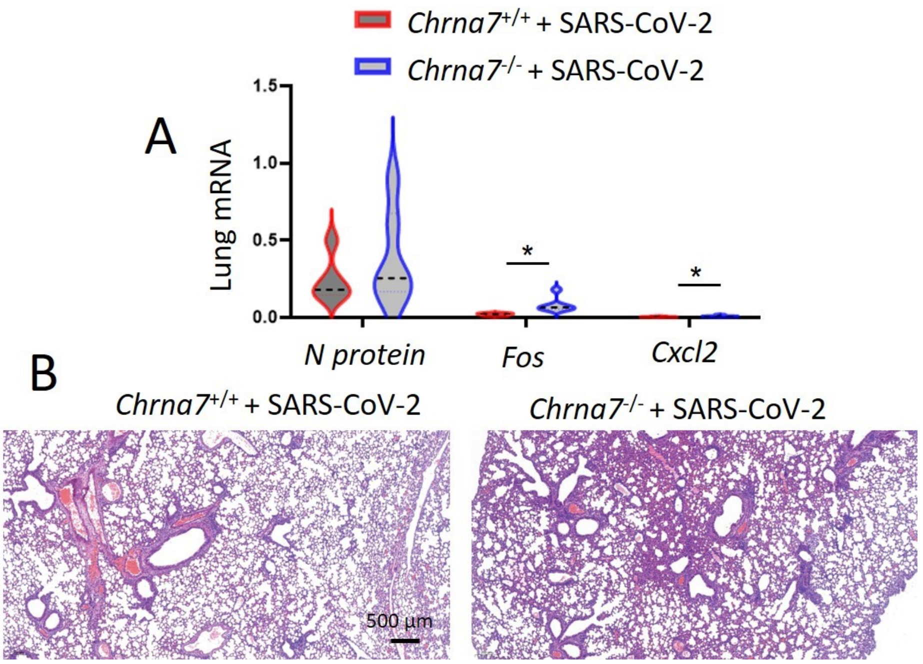

Supplementary Figure S8. Deficiency of *Chrna7* affects lung inflammation in SARS-CoV-2 infected mice. The mice were infected with SARS-CoV-2 for 4 days and sacrificed.

The lungs were removed to extract RNA for qPCR or histology. **A** Changes of lung SRARs-CoV-2 *N protein gene*, *Fos*, and *Cxcl2*. N = 5 in each group. *P < 0.05, unpaired t test. **B** Histology of SARS-CoV-2-infected lungs from both *Chrna7* wildtype and knockout mice. (H&E staining, objective magnification × 10, scale bar 500 μm); N = 5 in each group.

Repeated experiment G

As stated in our last response to a reviewer request, we are unable to replicate SARS-Cov-2 infection experiments due to the temporary unavailability of the P3 facility. However, in the pseudo-SARS-Cov-2-infected Calu3 cells, we found that both pretreatment and treatment with GTS-21 reduced ACE2 and FOS expression (A and B).

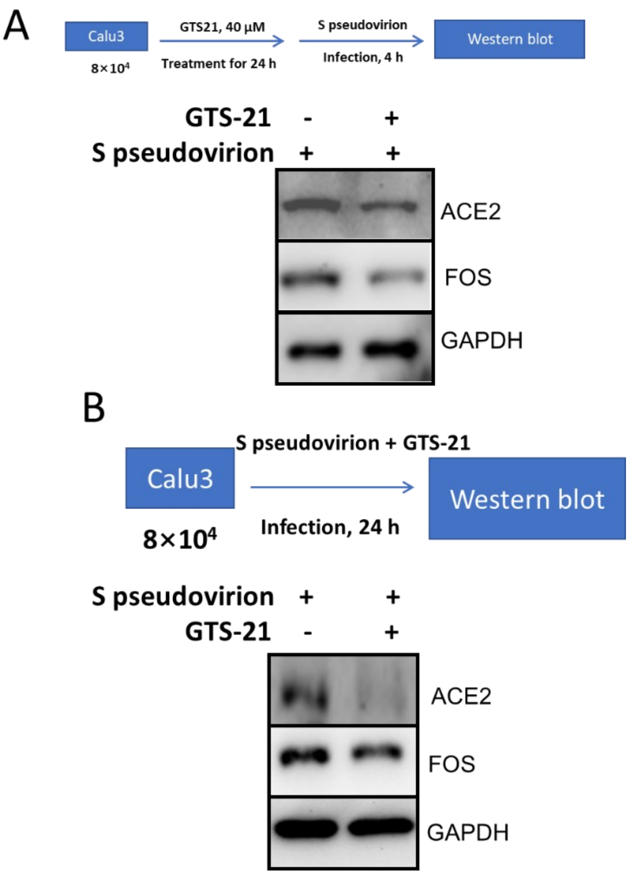

Repeated experiment H

T-5224 is a transcription factor c-Fos/AP-1 inhibitor, which specifically inhibits the DNA binding activity of c-Fos/c-Jun without affecting other transcription factors. As below figure demonstrated that treatment with T5224 in Caco2 cells (A) reduced ACE2

expression at both mRNA and protein levels (B-C).

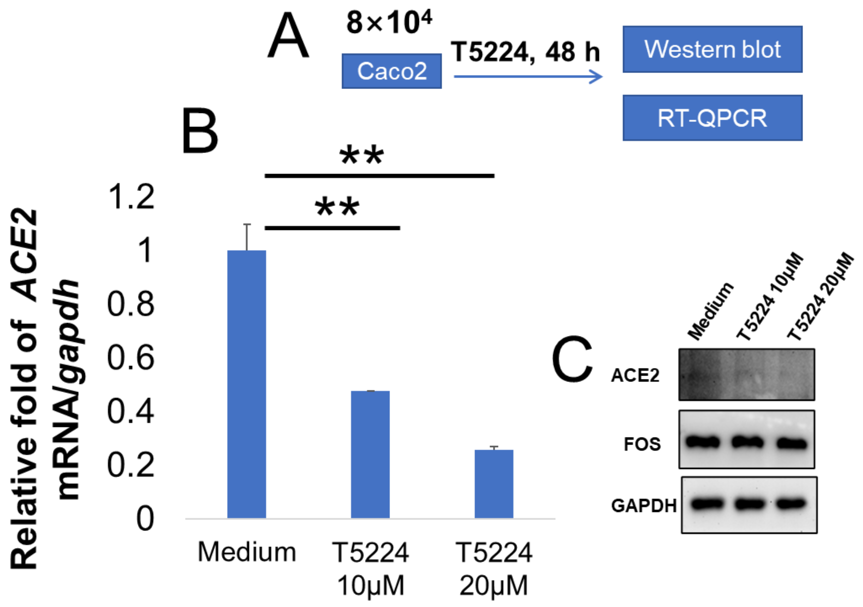

Supplement: mjad048_Supplemental_File [file mjad048_supplemental_file.pdf]
